# Identification of potential genetic components involved in the deviant quorum-sensing signaling pathways of *Burkholderia glumae* through a functional genomics approach

**DOI:** 10.3389/fcimb.2015.00022

**Published:** 2015-03-10

**Authors:** Ruoxi Chen, Inderjit K. Barphagha, Jong Hyun Ham

**Affiliations:** Department of Plant Pathology and Crop Physiology, Louisiana State University Agricultural CenterBaton Rouge, LA, USA

**Keywords:** *Burkholderia glumae*, bacterial panicle blight of rice, bacterial grain rot of rice, quorum-sensing, toxoflavin

## Abstract

*Burkholderia glumae* is the chief causal agent for bacterial panicle blight of rice. The acyl-homoserine lactone (AHL)-mediated quorum-sensing (QS) system dependent on a pair of *luxI* and *luxR* homologs, *tofI* and *tofR*, is the primary cell-to-cell signaling mechanism determining the virulence of this bacterium. Production of toxoflavin, a major virulence factor of *B. glumae*, is known to be dependent on the *tofI/tofR* QS system. In our previous study, however, it was observed that *B. glumae* mutants defective in *tofI* or *tofR* produced toxoflavin if they grew on the surface of a solid medium, suggesting that alternative signaling pathways independent of *tofI* or *tofR* are activated in that growth condition for the production of toxoflavin. In this study, potential genetic components involved in the *tofI*- and *tofR*-independent signaling pathways for toxoflavin production were sought through screening random mini-Tn*5* mutants of *B. glumae* to better understand the intercellular signaling pathways of this pathogen. Fifteen and three genes were initially identified as the potential genetic elements of the *tofI*- and *tofR*-independent pathways, respectively. Especially, the ORF (bglu_2g06320) divergently transcribed from *toxJ*, which encodes an orphan LuxR protein and controls toxoflavin biosynthesis, was newly identified in this study as a gene required for the *tofR-*independent toxoflavin production and named as *toxK*. Among those genes, *flhD*, *dgcB*, and *wzyB* were further studied to validate their functions in the *tofI-*independent toxoflavin production, and similar studies were also conducted with *qsmR* and *toxK* for their functions in the *tofR-*independent toxoflavin production. This work provides a foundation for future comprehensive studies of the intercellular signaling systems of *B. glumae* and other related pathogenic bacteria.

## Introduction

*Burkholderia glumae* causes bacterial panicle blight of rice and produces major virulence factors, such as toxoflavin and lipase, under the control of the quorum sensing (QS) system mediated by the *luxI* homolog, *tofI*, and the *luxR* homolog, *tofR* (Kim et al., 2004, 2007; Devescovi et al., 2007). Kim et al. (2004) used biosensors and thin layer chromatography to determine the acyl-homoserine lactone (AHL)-type autoinducers of *B. glumae* and found that *N*-octanoyl homoserine lactone (C8-HSL) and *N-*hexanoyl homoserine lactone (C6-HSL) are produced by the LuxI homolog, TofI. C8-HSL is considered as the functional autoinducer binding to TofR for promoting the virulence-related phenotypes including toxoflavin production and flagellum-mediated motility, while the role of C6-HSL is still vague (Kim et al., 2004, 2007). Several important genes for the virulence of *B. glumae* regulated by C8-HSL and its cognate receptor TofR include: the *tox* gene clusters (operons) for toxoflavin biosynthesis (*toxABCDE*) and transport (*toxFGHI*) (Kim et al., 2004; Shingu and Yoneyama, 2004; Suzuki et al., 2004), *lipA* encoding the LipA lipase (Devescovi et al., 2007), genes for flagellar biogenesis and *qsmR* encoding an IclR-type transcriptional regulator (Kim et al., 2007), and *katG* encoding a protective catalase (Chun et al., 2009). In addition, genes for the type III secretion system (T3SS) was demonstrated to be a part of the regulon of the *tofI/tofR* QS system (Kang et al., 2008). As these genes under the control of the *tofI/tofR* QS is important for the survival, colonization and pathogenesis of *B. glumae*, it will be beneficial to expand the knowledge upon the intercellular signaling network involving *tofI* and *tofR* for gaining a better understanding of the pathogenic behaviors of this pathogen.

In our previous study, a series of deletion mutants of *B. glumae* for *tofI* and *tofR* were generated for comprehensive characterization of the *tofI/tofR* QS system, using a Louisiana strain of *B. glumae*, 336gr-1 (Chen et al., 2012). Consistent with the previous studies with mutant derivatives of the *B. glumae* strain BGR1, *ΔtofI* or *ΔtofR* derivatives of *B. glumae* 336gr-1 did not produce toxoflavin in Luria-Bertani (LB) broth (Chen et al., 2012). However, these mutants produced high levels of toxoflavin when they were grown on solid media, including Luria broth (LB) agar and King's B (KB) agar (Chen et al., 2012). These results indicate the presence of previously unknown signaling/regulatory pathways for the production of toxoflavin that are activated in certain growth conditions (e.g., solid media) in *tofI*- and *tofR*-independent manners (Chen et al., 2012). In this study, *ΔtofI* and *ΔtofR* derivatives of *B. glumae* 336gr-1 were randomly mutagenized with mini-Tn*5 Cm* and the mutants showing lost ability of toxoflavin production on LB agar were screened in an attempt to identify and characterize the *tofI-* and *tofR-*independent signaling/regulatory pathways for toxoflavin production. For more sensitive visual detection of altered toxoflavin production by each transposon mutant, a DNA construct that harbors the promoterless *gusA* reporter gene fusion to the promoter region of the toxoflavin biosynthesis operon *toxABCDE*, *P_toxABCDE_*::*gusA*, was introduced into the *ΔtofI* and *ΔtofR* mutants, and the expression of the fused *gusA* reporter gene in each mutant was monitored based on the blue coloration of each mutant on LB agar containing the substrate of β-glucuronidase, 5-bromo-4-chloro-1*H*-indol-3-yl β-D-glucopyranosiduronic acid (X-gluc). Mutated genes of the primarily screened mutants, which did not show blue coloration on LB agar with X-gluc, were identified by sequencing the flanking regions of mini-Tn*5 Cm* inserted in the genome and the functions of selected genes were further studied in terms of their regulatory roles in toxoflavin production and virulence.

## Materials and methods

### Growth conditions of bacterial strains

The bacterial strains and plasmid constructs used in this study are listed in Table 1. Media used for routine cultures of bacterial strains were LB broth or LB agar (Sambrook, 2001) with appropriate amendments of antibiotics. Liquid cultures were incubated in a shaking incubator at 200 rpm at 37°C. The antibiotics and their working concentrations used in this study were: ampicillin (Amp), 100 μg/ml; kanamycin (Km), 50 μg/ml; nitrofurantoin (Nit), 100 μg/ml; and gentamycin (Gm), 20 μg/ml. The substrate of β-glucuronidase, X-gluc), was applied at a working concentration of 2 mM.

**Table 1:** **Bacterial strains and plasmids used in this study**.

**Strain or Plasmid**	**Description**	**References**
***Escherichia coli***		
DH10B	F^−^ *araD139* Δ(*ara, leu*)7697 Δ*lacX74 galU galK rpsL deoR* ø80d*lacZ*ΔM15 *endA1 nupG recA1 mcrA* Δ(*mrr hsdRMS mcrBC*)	Grant et al., 1990
DH5α	F^−^ *endA1 hsdR17* (r^−^_k_, m^+^_k_) *supE44 thi-1 λ^−^ recA1 gyrA96 relA1 deoR* Δ(*lacZYA*-argF)-U169 ø80d*lacZ*ΔM15	Grant et al., 1990
S17-1λ pir	*recA thi pro hsdR* [res− mod+][RP4::2-Tc::Mu-Km::Tn*7*] λ *pir* phage lysogen, Sm^*r*^/Tp^r^	Simon et al., 1983
***Burkholderia glumae***		
336gr-1	Wild type strain and the causative isolate of bacterial panicle blight of rice in Crowley, LA	This study
LSUPB22	spontaneous mutant of 336gr-1	This study
LSUPB145	A Δ*tofI* derivative of 336gr-1	Chen et al., 2012
LSUPB169	A Δ*tofR* derivative of 336gr-1	Chen et al., 2012
LSUPB139	A Δ*tofI-tofR* derivative of 336gr-1	Chen et al., 2012
LSUPB286	A Δ*tofM* derivative of 336gr-1	Chen et al., 2012
LSUPB172	A derivative of 336gr-1 carrying pKGpToxA-GUS	This study
LSUPB178	A derivative of Δ*tofI* carrying pKGpToxA-GUS	This study
LSUPB324	A derivative of Δ*tofR* carrying pKGpToxA-GUS	This study
LSUPB503	A *flhD^−^* derivative of the Δ*tofI* strain, LSUPB145	This study
LSUPB273	A *qsmR^−^* derivative of the wild type strain, 336gr-1	This study
LSUPB275	A *qsmR^−^* derivative of the Δ*tofI* strain, LSUPB145	This study
LSUPB277	A *qsmR^−^* derivative of the Δ*tofR* strain, LSUPB169	This study
LSUPB445	A *toxK^−^* derivative of the Δ*tofR* strain, LSUPB169	This study
LSUPB460	A *dgcB^−^* derivative of the wild type strain, 336gr-1	This study
LSUPB462	A *dgcB^−^* derivative of the Δ*tofI* strain, LSUPB145	This study
LSUPB464	A *dgcB^−^* derivative of the Δ*tofM* strain, LSUPB286	This study
LSUPB515	A *wzyB^−^* derivative of the Δ*tofI* strain, LSUPB145	This study
LSUPB516	A *wzyB^−^* derivative of the Δ*tofR* strain, LSUPB169	This study
***Chromabacterium violaceum***		
*Chromabacterium violaceum* CV026	A biosensor that can detect AHL molecules	McClean et al., 1997
**Plasmid**		
pSC-A-amp/kan	A blunt PCR cloning vector; f1 *ori*, pUC *ori*, *lacZ*', Km^R^, Amp^R^	Stratagene
pRK2013::Tn*7*	A helper plasmid; ColE1 *ori*	Ditta et al., 1980
mini-Tn*5 Cm*	A derivative of mini-Tn5 transposon, R6K *ori*, RP4 *oriT*, Cm^R^	De Lorenzo et al., 1990
pKNOCK-Km	A suicide vector; R6K *ori*, RP4 *oriT*, Km^R^	Alexeyev, 1999
pKNOCK-Gm	A suicide vector; R6K *ori*, RP4 *oriT*, Gm^R^	Alexeyev, 1999
pBBR1MCS-2	A broad host range cloning vector, RK2 *ori*, *lacZα*, Km^R^	Kovach et al., 1995
pBB2GUS	a derivative of pBBR1MCS-2 containing a promoterless *gusA*	This study
pSC-A-ptoxA	A PCR clone of 682 bp upstream promoter and partial coding region of *toxA* in pSC-A-amp/kan, Amp^R^, Km^R^	This study
pBB2GUS-ptoxA	A subclone of pSC-A-ptoxA for the promoter and partial coding region of *toxA* in pBB2GUS at *Hin*dIII site	This study
pKGpToxA-GUS	A subclone of pBB2GUS-ptoxA for the promoter and partial coding region of *toxA* gene and *gusA* gene in pKNOCK-Gm at *Kpn*I and *Spe*I sites	This study
PSC-*qsmR*	A PCR clone of 355-bp internal region of *qsmR* gene in pSC-A-amp/kan, Amp^R^, Km^R^	This study
PSC-flhD	A PCR clone of 304-bp internal region of *flhD* gene in pSC-A-amp/kan, Amp^R^, Km^R^	This study
PSC-toxK	A PCR clone of 402-bp internal region of *toxK* gene in pSC-A-amp/kan, Amp^R^, Km^R^	This study
PSC-dgcB	A PCR clone of 376-bp internal region of *dgcB* gene in pSC-A-amp/kan, Amp^R^, Km^R^	This study
PSC-wzyB	A PCR clone of 370-bp internal region of *wzyB* gene in pSC-A-amp/kan, Amp^R^, Km^R^	This study
pKKm*qsmR*	A subclone of pSC-*qsmR* for the internal region of *qsmR* gene in pKNOCK-Km at *Kpn*I and *Sac*II sites, Km^R^	This study
pKKmflhD	A subclone of pSC-flhD for the internal region of *flhD* gene in pKNOCK-Km at *Eco*RI site, Km^R^	This study
pKKmtoxK	A subclone of pSC-toxK for the internal region of *toxK* gene in pKNOCK-Km at *Eco*RI site, Km^R^	This study
pKGmdgcB	A subclone of pSC-dgcB for the internal region of *dgcB* gene in pKNOCK-Km at *Spe*I and *Hin*dIII sites, Km^R^	This study
pKGmwzyB	A subclone of pSC-wzyB for the internal region of *wzyB* gene in pKNOCK-Gm at *Spe*I and *Kpn*I sites, Km^R^	This study

### Recombinant DNA techniques

General DNA manipulations were conducted following standard methods (Sambrook, 2001). A Strata Clone™ PCR cloning kit (Agilent Technologies, Santa Clara, CA, USA) was used for cloning PCR products into a pSC-A-amp/kan vector. Cloned PCR products in pSC-A-amp/kan were sequenced for confirmation at GeneLab in the LSU School of Veterinary Medicine or at Macrogen USA (http://www.macrogenusa.com) using M13 forward and reverse primers. A GenePulser unit (BioRad Laboratories, Hercules, CA, USA) was used to perform electroporation for transforming *E. coli* competent cells under 1.5 kV, with 1 μl of ligated DNA and 25 μl of competent cells. Triparental mating was used for the transformation of *B. glumae* with DNA constructs (Figurski and Helinski, 1979). Digested DNA fragments were extracted from the agarose gel using GenElute™ Gel extraction kits (Sigma-Aldrich, St. Louis, MO, USA). The concentrations of the purified DNA and RNA were measured using a NanoDrop DN-1000 Spectrophotometer (Thermo Scientific, Wilmington, DE, USA). All the restriction enzymes used in this study were purchased from New England Biolabs (Beverly, MA, USA). The genomic library used in this study was constructed previously in our laboratory (Karki et al., 2012a).

### Construction of pKGpToxA-GUS

To generate the DNA construct for a *gusA* fusion to the promoter region of *toxABCDE* (*P_toxABCDE_*::*gusA*), the DNA fragment containing 562 bp upstream and 108 bp continuous coding sequences of *toxA* was amplified with the primer set, *toxA* PF/*toxA* PR (Table 2). The resultant PCR product was initially cloned into pSC-A-amp/kan, generating pSC-A-p*toxA*. The cloned region was then introduced into pBB2GUS, a broad host vector containing a promoterless *gusA*, using the unique *Hin*dIII site to obtain pBB2GUS-p*toxA*. The region containing the *P_toxABCDE_*::*gusA* fusion was cut with *Kpn*I and *Spe*I and ligated to the suicide vector, pKNOCK-Gm (Alexeyev, 1999), to generate pKGpToxA-GUS. This DNA construct was then introduced into the Δ*tofI* strain, LSUPB145, and the Δ*tofR* strain, LSUPB289, through tri-parental mating, generating LSUPB178 and LSUPB324, respectively.

**Table 2:** **The PCR programs and primers used for directional mutation**.

**Amplified region**	**Product length**	Primers^*^ (5′ 3′)	**PCR program**
Promoter and partial coding region of *toxA*	682 bp	toxA PF: AAGCTTTCCCTTCGCTTTTC (*Hin*dIII) toxA PR: CTCGAGACCAATCATGTGGAA (*Xho*I)	Annealing at 55°C Extension for 1 min
Flanking region of miniTn*5 Cm* insertion	Variable	Y Linker Primer: CTGCTCGAATTCAAGCTTCT Tn5 primer: GGCCAGATCTGATCAAGAGA	Annealing at 58°C Extension for 1 min Kwon and Ricke, 2000
Internal region for *qsmR*	355 bp	*qsmR*IK FP: CCGCCTCGGTGCTCGAACTG *qsmR*IK RP: AGCGTATCCTCCAGGGCGGG	Annealing at 60°C Extension for 1 min
Internal region of *flhD*	304 bp	FlhD-1: AATGCTCGCCGAGATCAA FlhD-2: TTAGCGGAGGCTTTCGAC	Annealing at 54°C Extension for 40 s
Internal region of *flhC*	462 bp	FlhC-1: GTGCTCGAGGTCAAGGAAATC FlhC-2: CAGCCCGCAGACGAAAC	Annealing at 54°C Extension for 40 s
Internal region of *toxK*	402 bp	OR-1: GATTCAGGCGGGCTAGTTT OR-2: CGCCGAATACGGCTACTG	Annealing at 54°C Extension for 40 s
Internal region of *dgcB*	376 bp	DGC-FP: CGTAGGTGTCGTTGTACTGCTTGA DGC-RP: ATCATCGTGCTGTCGACCAAGGA	Annealing at 58°C Extension for 30 s
Internal region of *wzyB*	370 bp	Oap-1: ACTCGCACGACATCTTCATC Oap-2: GGGTTCGTGCCGTAATAGAG	Annealing at 53°C Extension for 30 s
Region spanning the potential suicide vector inserted sites in *flhD*	445 bp	FLHD-C1: GCCACAATGACTGCAAGAATATAA FLHD-C2: GCAGATGATGTAGGGAGTGTTAG	Annealing at 53°C Extension for 30 s
Region spanning the potential suicide vector inserted sites in *toxK*	683 bp	toxK-C1: GGCAGCAAATCTCCGTTTATTC toxK-C2: GTACCGGTGCTGGATATGATT	Annealing at 54°C Extension for 45 s
Region spanning the potential suicide vector inserted sites in *qsmR*	863 bp	*qsmR*-C1: CCAGCGTGGACTTTGTCAT *qsmR*-C2: CAGTCTCGAGCAGCCATTC	Annealing at 52.5°C Extension for 1 min
Region spanning the potential suicide vector inserted sites in *dgcB*	878 bp	DGCB-C1: ATTGCGCATTCTGAAGGAAAC DGCB-C2: CAGCACGACACCGAACT	Annealing at 55°C Extension for 1 min
Region spanning the potential suicide vector inserted sites in *wzyB*	818 bp	Oap-C1: TGCACTATCACCTCGGTCT Oap-C2: CCAGTCGTGCAGTTCCTC	Annealing at 55°C Extension for 1 min

* *The restriction sites added in the primers are underlined*.

### Random mutation of *B. glumae* and screening of random *B. glumae* mutants

Mini-Tn*5 Cm*, a mini-Tn*5* transposon carrying a Cm resistant gene (De Lorenzo et al., 1990), was introduced to LSUPB178 (a Δ*tofI* derivative carrying the *P_toxABCDE_*::*gusA* fusion in the genome) and LSUPB324 (a Δ*tofR* derivative carrying the *P_toxABCDE_*::*gusA* fusion in the genome) by tri-parental mating for random mutagenesis. After the tri-parental mating, mini-Tn*5 Cm* mutants that did not show blue coloration (indicating no β-glucuronidase gene activity) on LB_Gm/Cm/Nir/X−gluc_ plates after 2 days of incubation at 37°C were collected for identifying the genes disrupted by mini-Tn*5 Cm*.

### Identification of genes disrupted by Mini-Tn5 Cm

The mutated genes of the screened mutants were identified following a previously developed method (Kwon and Ricke, 2000) with some modifications. Briefly, genomic DNA of mutant strains were extracted using a GenElute™ Bacterial Genomic DNA kit (Sigma-Aldrich, St. Louis, MO, USA). Genomic DNA (ca. 4 mg) was digested with two restriction enzymes, PstI and NlaIII, and the digested DNA fragments were ligated to the Y-shaped linker. Then, Y Linker Primer, CTGCTCGAATTCAAGCTTCT (specific to Y linker sequence), and Tn5 primer, GGCCAGATCTGATCAAGAGA (specific to transposon), were used to amplify the DNA sequences flanking where the transposon was inserted. The amplified PCR products were sequenced and the sequence data were BLAST-searched against the National Center for Biotechnology Information (NCBI) database for identification of the sequenced region.

### Directional mutation for validation of gene functions

A 300—400 bp internal DNA sequence of a gene of interest was amplified using a primer set indicated in Table 2 and cloned into a pKNOCK suicide vector, pKNOCK-Km or pKNOCK-Gm (Alexeyev, 1999). Specifically, PCR products for 355 bp of *qsmR*, 304 bp of *flhD*, 402 bp of *toxK*, 376 bp of *dgcB* and 370 bp of *wzyB* were cloned into PSC-A-amp/kan to generate PSC-*qsmR*, PSC-flhD, PSC-toxK, PSC-dgcB and PSC-wzyB, respectively.*Kpn*I and *Sac*II sites were used to transfer the *qsmR* region in PSC-*qsmR* to pKNOCK-Km to generate pKKm*qsmR*. *EcoR*I site was used to move the *flhD* and *toxK* regions in PSC-flhD and PSC-toxK to pKNOCK-Km vector, generating pKKmflhD and pKKmtoxK, respectively. *Spe*I and *Hin*dIII were used to transfer the *dgcB* region from pSC-dgcB to pKNOCK-Gm, generating pKGmdgcB. *Spe*I and *Kpn*I were used to transfer the *wzyB* region from pSC-wzyB to pKNOCK-Gm, generating pKGmwzyB. Each final DNA construct in a pKNOCK suicide vector was introduced into *B. glumae* strains by tri-parental mating. Diagnostic PCR was conducted to confirm if the internal sequence of each gene was successfully integrated to the bacterial genome through homologous recombination. Primer pairs listed in Table 2, FLHD-C1/FLHD-C2, toxK-C1/toxK-C2, *qsmR*-C1/*qsmR*-C2, DGCB-C1/DGCB-C2, and Oap-C1/Oap-C2, were used for the diagnostic PCRs to confirm the targeted mutations of *flhD*, *toxK*, *qsmR*, *dgcB*, and *wzyB*, respectively.

### Quantification of toxoflavin production by *B. glumae* strains grown on LB agar

Toxoflavin produced by *B. glumae* strains grown on LB agar was quantified following a previous method (Chen et al., 2012) with miscellaneous modifications. Briefly, overnight bacterial cultures were concentrated to OD_600_ = 100 (~10^11^ cfu/ml) and one loopful of each inoculum was streaked on the entire area of a LB agar plate. After 24 h incubation at 37°C, the bacterial culture was scraped off from the surface of the LB agar. Five grams of the LB agar where bacteria were grown on was then cut into small pieces with a razor blade. The chopped agar pieces were mixed with 5 ml chloroform and left to sit at room temperature for 30 min. The chloroform fraction was then transferred to a new tube and air-dried under a fume hood. The remaining materials in the dried tube were dissolved in 1 ml of 80% methanol. The OD_393*nm*_ values were measured to determine the quantity of toxoflavin, using a spectrophotometer. The sample from uncultured LB agar was used to set the zero of OD_393*nm*_.

### Virulence assay with onion bulb scales

Virulence tests for the mutant strains of *B. glumae* were conducted using onion bulbs as a surrogate host, following a previously described method (Karki et al., 2012b).

## Results

### Fifteen protein-coding genes were identified as potential genetic components for the tofi-independent production of toxoflavin

Both LSUPB178 (a Δ*tofI* derivative carrying the *P_toxA_*::*gusA* fusion in the genome) and LSUPB324 (a Δ*tofR* derivative carrying the *P_toxA_*::*gusA* fusion in the genome) showed blue coloration on LB agar containing X-gluc, indicating that the introduced *gusA* fusion to the *toxA* promoter is functional (data not shown). After the random mutagenesis of LSUPB178 with mini-Tn*5 Cm*, the mutant colonies that did not exhibit blue color were picked from the selection plates (LB agar amended with Gm, Cm, Nit, and X-gluc). It was considered that mini-Tn*5 Cm* is inserted in a potential genetic component of the *tofI-*independent toxoflavin production pathway in the mutants screened. Among the 4400 random mini-Tn*5 Cm* derivatives of LSUPB178 observed, 16 mutant derivatives were initially screened and their mutated genes were identified through BLAST searches of the flanking sequences (Table 3). All the 16 mutant strains screened were confirmed to be toxoflavin-deficient on LB agar at 37°C (data not shown). Fifteen out of the 16 mutants screened turned out to have mini-Tn*5 Cm* insertion in protein-coding regions and one mutant had the insertion in the middle of a 23S ribosomal RNA sequence. The 15 protein-coding genes identified include *toxR* and *toxA*, which are known to be required for toxoflavin production (Herrmann and Weaver, 1999), indicating the validity of this experiment (Table 3). Among the 15 genes identified, three genes (*flhD* encoding a transcriptional activator for flagellar biogenesis, *dgcB* encoding a putative diaguanylate cyclase, and *wzyB* encoding a putative O-antigen polymerase family protein) were selected for the extended functional studies described below.

**Table 3:** **The list of potential genes contributing to the *tofI-*independent toxoflavin production**.

**Name of random mutants**	**Locus of inserted genes**	**Function of disrupted genes**
LSUPB186	bglu_1g10100	Succinylornithine transaminase
LSUPB187	bglu_2g10840^*^	putative LysM domain-containing protein
LSUPB182	bglu_1g02180	Diguanylate cyclase
LSUPB183	bglu_1g01780^*^	Flagellar transcriptional activator FlhD
LSUPB184	bglu_2g07160^*^	Catechol 1,2-dioxygenase
LSUPB185	bglu_1g00380	General secretory pathway protein D
LSUPB188	bglu_2g22000^*^	Hypothetical protein
LSUPB189	bglu_1g07800	Hypothetical protein
LSUPB192	bglu_2g06390	LysR family transcriptional regulator (*toxR*)
LSUPB193	bglu_2g06400^*^	putative ubiquinone/menaquinone biosynthesis methyltransferase (*toxA*)
LSUPB194	bglu_2g18120	Amylo-alpha-1,6-glucosidase family protein
LSUPB209	bglu_1g33070	Flagellar hook-associated protein FlgK
LSUPB210		rRNA-23S ribosomal RNA
LSUPB211	bglu_1g29900	O-antigen polymerase family protein
LSUPB212	bglu_1g00440	Glutamate–cysteine ligase
LSUPB213	bglu_1g05190	Short chain dehydrogenase

* *Mini-Tn5Cm is inserted in the promoter region*.

### Three protein-coding genes were identified as potential genetic components for the tofR-independent production of toxoflavin

Similar to LSUPB178, LSUPB324 (Δ*tofR*:: *P_toxABCDE−gusA_*) exhibited blue color on LB agar X-gluc (data not shown). Three mini-Tn*5 Cm* derivatives of LSUPB324 that did not produce blue color were characterized for the mutated genes (Table 4), and loss of the function in toxoflavin production was observed with all the three mutants (data not shown). Particularly, one toxoflavin-deficient mini-Tn*5 Cm* mutant, LSUPB191, turned out to be disrupted in the ORF, bglu_2g06320 (Table 4). This ORF is located upstream of and divergently transcribed from *toxJ*, a known regulatory gene for toxoflavin biosynthesis encoding an orphan LuxR protein. Due to its apparent function in toxoflavin production, the ORF was newly named as *toxK* in this study. Among the three identified genes, *qsmR* and *toxK* were chosen for the further functional studies described below.

**Table 4:** **The list of *tofR-*independent genes contributing to *tofR-*independent toxoflavin production**.

**Name of random mutants**	**Locus of inserted genes**	**Function of disrupted genes**
LSUPB190	bglu_1g10250	IclR family regulatory protein gene (*qsmR*)
LSUPB191	bglu_2g06320	Hypothetical protein in the upstream of *toxJ* encoding an orphan LuxR family transcriptional regulator (named as *toxK* in this study)
LSUPB195	bglu_2g22590	Putative PAS/PAC sensor protein

### Targeted mutation of *flhD*, *dgcB*, *wzyB*, *qsmR*, or *toxK* resulted in impaired toxoflavin production

To confirm the initially observed functions of *flhD*,*dgcB*, and *wzyB* in *tofI-*independent toxoflavin production, independent mutants of these genes were generated via homologous recombination. For this, internal regions of *flhD*,*dgcB*, and *wzyB* were cloned in a suicide vector (pKNOCK-Km or pKNOCK-Gm) and introduced into the *ΔtofI* background, LSUPB145, generating the corresponding mutants, LSUPB503, LSUPB462, and LSUPB515, respectively (see the Materials and Methods section for detail). The same strategy was applied for the targeted mutation of *qsmR* and *toxK* in the *ΔtofR* background, LSUPB169, generating the corresponding mutants LSUPB277 and LSUPB445, respectively. These independent mutants of the five genes were then examined for their phenotypes in toxoflavin production in comparison with their parental strains.

#### flhD

**Figure 1:**
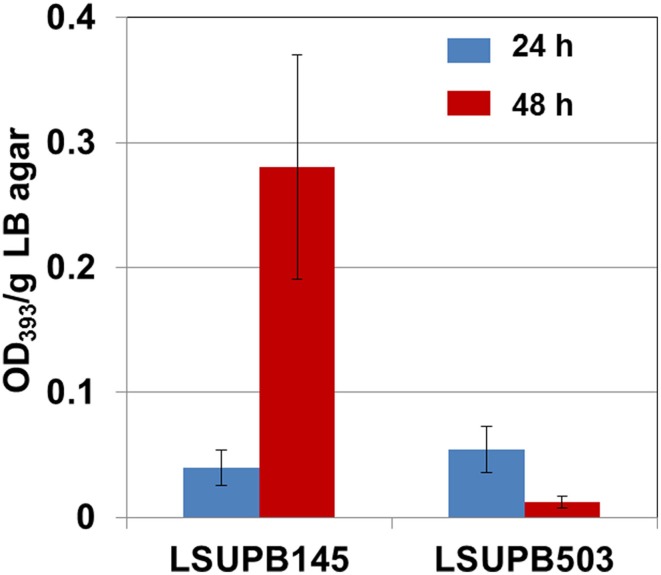
**Toxoflavin production of LSUPB503 (*flhD^−^/*Δ *tofI*) compared to its parental strain, LSUPB145 (Δ *tofI*), grown on LB agar after 24 and 48 h of incubation at 37°C**. The amounts of toxoflavin are indicated with the OD_393_/g LB agar values.

Compared to LSUPB145, its *flhD^−^* derivative LSUPB503 showed a substantial reduction in toxoflavin production (Figure 1). There was little difference between LSUPB145 and LSUPB503 during the first 24 h of incubation period, but during the additional 24 h incubation period, reduction of toxoflavin amount was observed with LSUPB503, while more than 5 times increase of toxoflavin was observed with LSUPB145 during the same incubation period (Figure 1). Null mutation of *flhC*, which is located downstream of *flhD*, in the *ΔtofI* background through homologous recombination of an internal coding region also caused loss of toxoflavin production (data not shown).

#### dgcB

**Figure 2:**
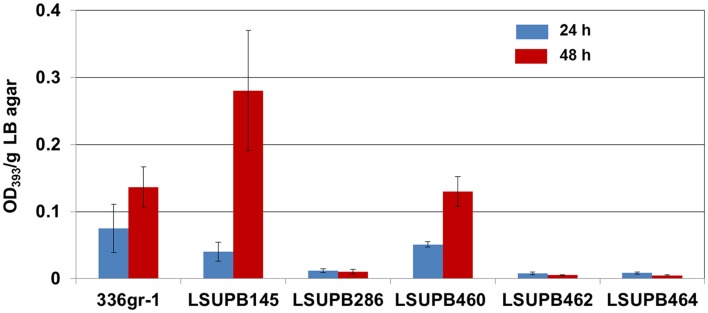
**Toxoflavin production of the *dgcB* mutants, LSUPB460 (*dgcB^−^*), LSUPB462 (*dgcB*^−^/Δ*tofI*) and LSUPB464 (*dgcB*^−^/Δ*tofM*), compared to their parental strains, 336gr-1 (wild type), LSUPB145 (Δ*tofI*) and LSUPB286 (Δ*tofM*), grown on LB agar after 24 and 48 h of incubation at 37°C**. The amounts of toxoflavin are indicated with the OD_393_/g LB agar values.

Both of the two independent *dgcB* null mutants, LSUPB462 (a *dgcB*::pKGmdgcB derivative of the *ΔtofI* strain LSUPB145) and LSUPB182 (a *dgcB*::mini-Tn*5 Cm* derivative of LSUPB145), were toxoflavin-deficient on LB agar medium in common (data not shown). This indicates the important role of the DgcB diguanylate cyclase in the *tofI-*independent pathway for toxoflavin production. To know if *dgcB* is also critical for toxoflavin production when the *tofI/tofR* QS system is intact, LSUPB460 (a *dgcB^−^* derivative of the wild type strain 336gr-1) was generated and examined for toxoflavin production. As shown in Figure 2, LSUPB460 produced a similar level of toxoflavin production compared to 336gr-1. This suggests that *dgcB* plays a positive role in toxoflavin production but this function is dispensable in the presence of the intact *tofI/tofR* QS system. To explore the relationship between *dgcB* and other QS elements *tofR* and *tofM* (Chen et al., 2012), pKGmdgcB was introduced into LSUPB169 (a *ΔtofR* mutant) and LSUPB286 (a *ΔtofM* mutant) for generating *dgcB^−^* derivatives of these QS mutant strains. But *dgcB^−^* derivatives could be obtained from only LSUPB286 but not LSUPB169, and the *dgcB^−^/ΔtofM* strain, LSUPB464, also showed a toxoflavin-deficient phenotype like its parent, LSUPB286 (Figure 2).

#### wzyB

**Figure 3:**
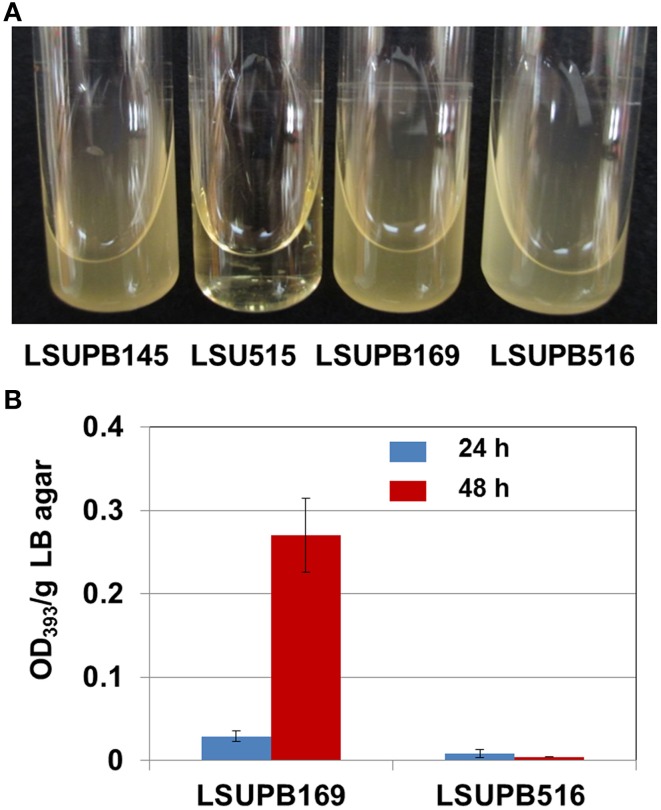
**Bacterial growth and toxoflavin production of *wzyB* mutants. (A)** Bacterial growth in LB broth at 37**°**C: LSUPB145 (Δ*tofI*), LSUPB515 (*wzyB^−^/*Δ*tofI*), LSUPB169 (Δ*tofR*) and LSUPB516 (*wzyB^−^/*Δ*tofR*). Photo was taken 48 h after inoculation. **(B)** Toxoflavin production of the *wzyB* mutant, LSUPB516 (*wzyB^−^/*Δ*tofR*), compared to its parental strains, LSUPB169 (Δ*tofR*), grown on LB agar after 24 and 48 h of incubation at 37°C. The amounts of toxoflavin are indicated with the OD_393_/g LB agar values.

LSUPB515, the *wzyB* disruptive mutant in LSUPB145 background grew very slowly on both LB agar (data not shown) and LB broth (Figure 3A). Because of the extremely slow growth of the bacterial cells, it was difficult to conduct reliable toxoflavin quantification for LSUPB515. To determine if *wzyB* is also required for the *tofR-*independent toxofalvin production, a disruptive mutant in LSUPB169 background, LSUPB516, was generated and tested for its ability to produce toxoflavin. LSUPB516 showed normal cell growth unlike LSUPB515 (Figure 3A) and a toxoflavin-deficient phenotype (Figure 3B), indicating that *wzyB* is required for both *tofI-* and *tofR-*independent toxoflavin production. Generation of a *wzyB* null mutant derivative of 336gr-1 to determine the role of this gene in the wild type background was not successful.

#### qsmR

**Figure 4:**
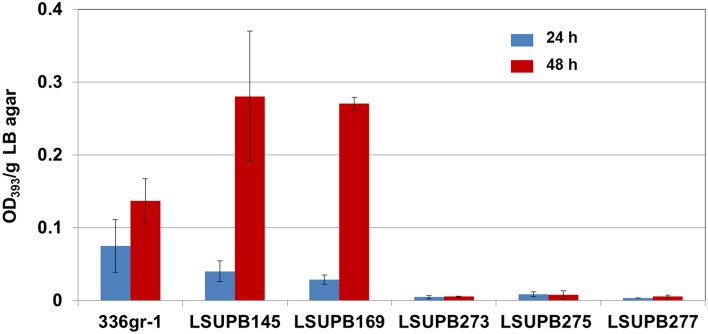
**Toxoflavin production of the *qsmR* mutants, LSUPB273 (*qsmR^−^*), LSUPB275 (*qsmR^−^/*Δ*tofI*) and LSUPB277 (*qsmR^−^/*Δ*tofR*), compared to their parental strains, 336gr-1 (wild type), LSUPB145 (Δ*tofI*) and LSUPB169 (Δ*tofR*), grown on LB agar after 24 and 48 h of incubation at 37°C**. The amounts of toxoflavin are indicated with the OD_393_/g LB agar values.

LSUPB277, *qsmR* mutated in LSUPB169 background, had the same toxoflavin loss phenotype as the random mutant LSUPB190, which confirmed the important role of *qsmR* in *tofR-*independent toxoflavin production (Figure 4). To determine the role of *qsmR* in toxoflavin production in the presence and absence of the intact QS system, LSUPB273 (a *qsmR* mutant in the wild type background 336gr-1) and LSUPB275 (a *qsmR* mutant in the Δ*tofI* background LSUPB145) were also generated and tested for their phenotypes in toxoflavin production. As shown in Figure 4, all the *qsmR* knock out mutants tested were deficient in toxoflavin production regardless of the genetic background of their parental strains, indicating the essential role of *qsmR* in the production of toxoflavin.

#### toxK

**Figure 5:**
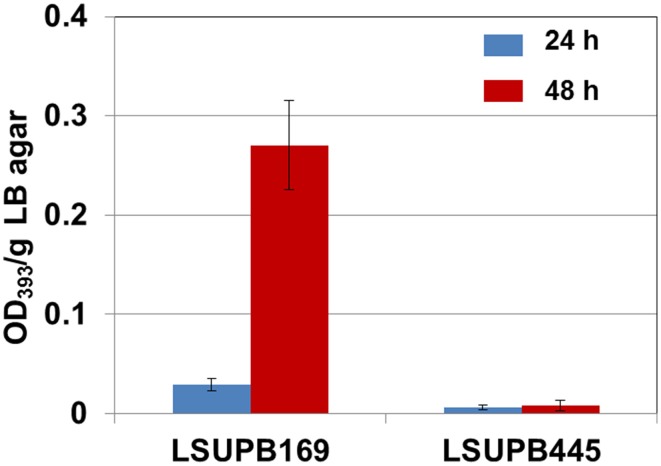
**Toxoflavin production of the *toxK* mutant LSUPB445 (*toxK^−^/*Δ*tofR*) compared to its parental strain, LSUPB169 (Δ*tofR*), grown on LB agar after 24 and 48 h of incubation at 37°C**. The amounts of toxoflavin are indicated with the OD_393_/g LB agar values.

As shown in Figure 5, toxoflavin production was almost abolished in the *toxK* mutant in LSUPB169 background, LSUPB445, indicating that this putative gene in front of *toxJ* encoding an orphan LuxR protein is essential for toxoflavin production of *B. glumae* in the absence of *tofR*. The function of *toxK* in the wild type and *ΔtofI* backgrounds has not been determined due to the failure of generation of *toxK^−^* and *toxK^−^/ΔtofI* strains through homologous recombination.

### Onion maceration caused by mutants

**Figure 6:**
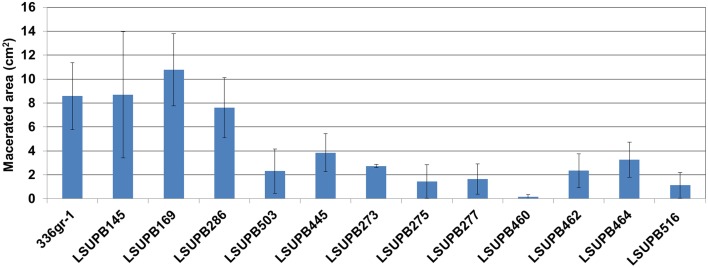
**Virulence of *Burkholderia glumae* 336gr-1 and its various mutant derivatives determined by the maceration areas they produced on onion bulb scales**. The two diameters of each maceration area (*a* and *b*) were measured to calculate the size of the area, using the formula: Area (cm^2^) = π*ab*. Each error bar indicates the standard deviation from four replications.

The virulence functions of the five selected genes were determined with the virulence assay system using onion bulb scales as a surrogate host (Karki et al., 2012a,b). The strains included in the virulence assay were; 336gr-1 (wild type), LSUPB145 (ΔtofI), LSUPB169 (ΔtofR), LSUPB286 (ΔtofM), LSUPB503 (flhD*^−^* in ΔtofI), LSUPB445 (toxK*^−^* in ΔtofR), LSUPB273 (*qsmR^−^*), LSUPB275 (*qsmR^−^* in ΔtofI), LSUPB277 (*qsmR^−^* in ΔtofR), LSUPB460 (dgcB*^−^*), LSUPB462 (dgcB*^−^* in ΔtofI), LSUPB464 (dgcB*^−^* in ΔtofM), and LSUPB516 (wzyB*^−^* in ΔtofR). As shown in Figure 6, all the mutants tested exhibited reduced virulence compared to their parental strains even though none of them were completely avirulent, indicating the positive roles of the five genes in bacterial pathogenesis.

## Discussion

### Genome-wide screening by random transposon mutagensis revealed novel regulators of toxoflavin in *B. glumae*

The *toxA* promoter and *gusA* transcriptional fusion was introduced to Δ*tofI* and Δ*tofR* mutants and random mutagenesis with mini-Tn*5 Cm* was implemented to search for the genes that could affect the expression of the *toxABCDE* operon, thus the toxoflavin biosynthesis, in *tofI-* or *tofR-*independent ways. Through this approach, fifteen and three protein-coding genes were identified as potential regulators for the *tofI-* and *tofR-*independent toxoflavin biosynthesis of *B. glumae* 336gr-1, respectively. Identification of *toxA* and *toxR*, which are known genes required for toxoflavin biosynthesis, verifies the reliability of this experiment. The reason for the abolishment of the *P_toxA::gusA_*expression by disruption of *toxA* may be due to the fact that *toxR*, which is an essential regulatory factor for toxoflavin biosynthesis, requires a residual amount of toxoflavin as a co-inducer for its functionality (Kim, 2004). Among the potential genetic elements for *tofI-* or *tofR-*independent toxoflavin production identified from screening of random mini-Tn*5 Cm* mutants, five genes (*flhD* encoding a flagella transcriptional activator, *dgcB* encoding a diguanylate cyclase, *wzyB* encoding an O-antigen polymerase family protein, *qsmR* encoding an IclR-type transcriptional regulator, and *toxK* encoding a hypothetical protein and divergently transcribed from *toxJ* encoding an orphan LuxR homolog essential for toxoflavin production) were further characterized in this study for their functions in toxoflavin production in the presence or absence of the intact TofI/TofR QS system.

Based on the annotated genome *B. glumae* BGR1 (Lim et al., 2009), it is unlikely that possible polar effects from the transposon or directional mutations can cause misinterpretation of the observed mutant phenotypes for the five genes investigated in this study. *flhD* (bglu_1g01780) and *flhC* (bglu_1g01790) comprise an operon, but their products are known to work together as one functional unit (Kim et al., 2007). In this study, both *flhD^−^* and *flhC^−^* mutants showed the toxoflavin-deficient phenotype in the *ΔtofI* background. In case of the other genes, *dgcB* (bglu_1g02180), *qsmR* (bglu_1g10250), and *toxK* (bglu_2g06320) are apparently monocistronic, while *wzyB* (bglu_1g29900) is located at the end of a putative operon structure containing three genes. Thus, polarity may not be a major concern with the five genes investigated in this study.

### *Flhd* is required for the toxoflavin production by *B. glumae* 336gr-1 in the Δ*tofI* background

The *flhDC* operon encoding the FlhDC complex was characterized as a flagellar transcriptional activator, which was controlled by QS in *B. glumae* BGR1 (Kim et al., 2007). It was reported that a *flhD* deficient mutant of BGR1 still produced toxoflavin but lost pathogenicity as well as flagellum-mediated motility (Kim et al., 2007). In this study with another strain of *B. glumae*, 336gr-1, it was observed that *flhD* as well as *flhC* were required for the production of toxoflavin in the absence of *tofI*. At this point, it has not been determined if *flhD^−^* or *flhC^−^* derivatives of 336gr-1 still produce toxoflavin like BGR1. Mutation of these genes in the wild type as well as in the*ΔtofR* backgrounds is currently under way. Due to the significant difference between BGR1 and 336gr-1 in the QS-mediated regulation of toxoflavin, it is possible that *flhDC* is not involved in toxoflavin production in BGR1. Nevertheless, this study indicates that *flhDC* has a regulatory function in toxoflavin production in addition to flagellum-mediated motilities in at least some strains of *B. glumae*.

### *dgcB* is required for toxoflavin production in the absence *tofI* but not in the wild type background having the intact *tofI/tofR* QS system

The results in this study indicate that *dgcB* is required for toxoflavin production in the absence of *tofI* but not in the presence of the intact TofI/TofR QS system. *dgcB* codes for a diguanylate cyclase, which synthesizes cyclic di-guanosine monophosphate (c-di-GMP) from two guanosine triphosphate (GTP) molecules. c-di-GMP is a small diffusible signal molecule that influences a wide range of cellular functions including bacterial virulence (Romling, 2012). The phenotype of the *dgcB* mutant, LSUPB462, observed in this study strongly implies that the c-di-GMP signaling plays a pivotal role in the regulation of virulence factors in *B. glumae*. Involvement of c-di-GMP signaling pathways for the pathogenesis by plant pathogenic bacteria has been studied mainly with *Xanthomonas* spp. Ham (2013). Regulatory functions of c-di-GMP for the expression of EPS and biofilm were recently reported with *B. cenocepacia* (Fazli et al., 2011, 2013; Deng et al., 2012). Even though this study adds to our knowledge about the function of c-di-GMP signaling in plant pathogenic bacteria, more studies including identification and functional characterization of other c-di-GMP signaling components, such as phosphodiesterases and c-di-GMP-binding protein, should be followed to clearly understand the role of this signaling system for the bacterial pathogenesis by *B. glumae*.

### *wzyB* encoding an O-antigen polymerase may play an important role in the *tofI*-independent intercellular signaling for toxoflavin production

*wzyB* was initially identified as a genetic element for the *tofI*-independent toxoflavin production through screening of random mini-Tn*5 Cm* mutant derivatives of the *ΔtofI* strain, LSUPB145. LSUPB515, an independent *wzyB* mutant derivative of LSUPB145 generated through homologous recombination, grew slowly due to an unknown reason and produced no observable toxoflavin. LSUPB516, a *wzyB* mutant derivative of the *ΔtofR* strain LSUPB169 through homologous recombination, also lost the ability to produce toxoflavin on a solid medium. These observations indicate that *wzyB* is required for both *tofI-* and *tofR-*independent toxoflavin production. However, it remains to be determined whether or not *wzyB* is also required for the toxoflavin production in the presence of the intact TofI/TofR QS system because *wzyB* mutation in the wild type background has been failed due to an unknown reason.

O-antigen is one of the three major components (the other two are lipid A and a core oligosaccharide) of lipopolysaccharides (LPS) in the outer membrane of Gram negative bacteria; and is structurally very diverse and highly immunogenic (Raetz and Whitfield, 2002; Stone et al., 2012). Study of O-antigen and its synthesis pathway can provide important antibiotic targets for potential clinical use. O-antigen polymerase family proteins (encoded by *wzy* homologs) are responsible for polymerizing the O-antigen units to O-antigen chain (Daniels et al., 1998; Chin et al., 2010). It is also well known that LPS is a common virulence determinant (Thomsen et al., 2003; Ellis and Kuehn, 2010). As an important synthesis enzyme for O-antigen and LPS, O-antigen polymerase should be important for maintaining the virulence of pathogenic bacteria. Even though not much study has been focused on *wzy* homologs, it was recently reported that non-polar mutation of the *wzy* gene in *Salmonella enterica* serovar *Typhimurium* caused attenuated virulence in mice (Kong et al., 2011). It would be an appealing hypothesis that the outer membrane-bound O-antigen produced by *wzy* genes mediates signal transduction dependent on “physical contact” of bacterial cells populated on a solid surface.

Furthermore, it would be worthy to make more investigation on the relationship between motility genes, especially *flhDC*, and *wzyB* as well as other LPS synthesis genes. There have been reports about how the subunits of and enzymes involved in assembling LPS influence the motility function of bacteria. The mutation of an O-antigen gene in *Salmonella enterica* serovar Typhimurium resulted in defective bacterial swarming and, in the same study, it was also suggested that the role of O-antigen is to improve the wettability of the bacterial colony (Toguchi et al., 2000). The mutation of *wzxE* gene, which is responsible for transporting O-antigen units across the inner membrane to the LPS assembling site (Chin et al., 2010), caused *E. coli* to lose both swimming and swarming motility (Girgis et al., 2007). The mutation of *waaL*, an O-antigen ligase gene, in *Vibrio fischeri* resulted in loss of motility and ability to survive with the wild type in the co-colony assay (Buttner, 2012). In another study, mutation of *waaL* blocked the expression of *flhDC* genes and aborted the swarming motility of human pathogen *Proteus mirabilis* in soft agar, but overexpression of *flhDC* genes in *trans* overcame this defect (Morgenstein et al., 2010). Therefore, motility assays are under way in our laboratory to determine the functional relationship between *wzyB* and the QS-regulated motility system in *B. glumae*.

### *qsmR* is an essential regulatory gene for the toxoflavin production of *B. glumae* 336gr-1

In this study, null mutation of *qsmR* resulted in lost function of toxoflavin production in the wild type (336gr-1), *ΔtofI* (LSUPB145) and *ΔtofR* (LSUPB169) backgrounds, indicating that *qsmR* is another essential regulatory factor for toxoflavin production in *B. glumae* 336gr-1. *qsmR* was previously reported as a positive regulator for *flhDC*, which was activated by C8-HSL and TofR complex in another strain of *B. glumae*, BGR1 (Kim et al., 2007). In the same study, disruption of *qsmR* resulted in loss of motility and pathogenicity in rice, but no observable difference in toxoflavin production in the solid medium condition (Kim et al., 2007). However, the *qsmR^−^* derivative of BGR1 showed decreased production and degradation of toxoflavin in the liquid medium condition from 12 h of incubation (Kim et al., 2007). In this study with *B. glumae* 336gr-1, the *qsmR* mutant (LSUPB273) did not produce observable toxoflavin on LB agar and still retained partial virulence on onion, which was somewhat different from the results of Kim et al. with *B. glumae* BGR1. These results from this and previous studies suggest that toxoflavin biosynthesis is regulated by *qsmR* more tightly in 336gr-1 than in BGR1. *qsmR* was also shown to regulate the *katG* catalase gene, which is required for the full virulence of *B. glumae* BGR1(Chun et al., 2009).

Additionally, recent studies with *B. glumae* BGR1 demonstrated that *qsmR* is a key player for both bacterial pathogenesis and survival in stressful conditions. From a proteomics search, the type II secretion system of *B. glumae* BGR1 encoded by *gsp* genes was found to be regulated by QS, and *qsmR* was shown to be a major positive regulator for *gsp* genes (Goo et al., 2010). In a study about how QS systems help representative *Burkholderia* species survive stationary phases, the sub-QS regulator *qsmR* was determined to be critical for activating the production of oxalate and maintaining normal pH levels of bacterial culture media (Goo et al., 2012). Without functional *qsmR*, there was a survival defect in *B. glumae* and *B. thailandensis* populations, which correlated with the high pH and lack of oxalate (Goo et al., 2012). Taken this and the previous studies on *qsmR* together, it is thought that *qsmR* plays an important role in general in the expression of various virulence factors in *B. glumae* despite some functional variations among different strains.

### Discovery of the *toxK* gene adds more complexity to the known regulatory cascade for toxoflavin biosynthesis involving the orphan luxR protein, *toxJ*

The ORF (gene ID of the reference sequence: bglu_2g06320) located upstream of and divergently transcribed from *toxJ*, the QS-dependent toxoflavin regulator encoding an orphan LuxR protein, was identified as a new genetic element required for the *tofR-*independent toxoflavin production of *B. glumae* 336gr-1 and named as *toxK*. There is one *lux* box like sequence (*tof* box) between *toxJ* and *toxK*, and it was previously proven that TofR and C8-HSL complex can bind on that site and activate the expression of *toxJ* (Kim et al., 2004). It may be the case that the *tof* box between *toxJ* and *toxK* promotes the expression of both genes, even though this notion should be proved experimentally. The deduced protein encoded by *toxK* contains 144 residues with 15.5 kDa of estimated molecular mass without an obvious homolog. Instead, the deduced protein (ID: ACR31068) has a conserved domain found in heterogeneous ribonucleoproteins (hnRNPs), implying that binding to target RNAs is its major functional mechanism.

It is interesting that the orphan *lux* homolog, *toxJ*, is next to an RNA-binding protein-coding gene instead of a *luxI* homolog. Regarding its location, size and predicted RNA-binding activity, *toxK* may be considered as a “chaperone” that modulates the expression and function of *toxJ*. More information about the function of *toxK* will be obtained when *toxK* mutants in the wild type and *ΔtofR* backgrounds are available and more phenotypic assays with *toxK* mutants in various genetic backgrounds are performed. In addition, future molecular studies to determine if mRNA of *toxJ* is a target of *toxK* and how *toxK* affect the expression of *toxJ* as well as other potential target genes will provide valuable insights into the functional basis of *toxJ* and, further, into a type of functional mechanism adopted by other LuxR solos.

In conclusion, potential genetic components involved in the deviant quorum-sensing signaling pathways of *B. glumae* for the production of the major virulence factor, toxoflavin, were identified in this study. In particular, the genes known for other functions, *flhDC*, *dgcB*, *wzyB*, and *qsmR*, as well as a previously unknown gene, *toxK*, were newly found to function in the *tofI-* and *tofR-*independent toxofalvin production via directional mutation of each gene. The information obtained from this study provides not only great insights into the complex cell-to-cell signaling network of this bacterium but a good foundation of future studies for comprehensive functional analyses of the individual genes identified in this study.

### Conflict of interest statement

The authors declare that the research was conducted in the absence of any commercial or financial relationships that could be construed as a potential conflict of interest.
